# Sociodemographic Variation in the Perception of Barriers to Exercise Among Japanese Adults

**DOI:** 10.2188/jea.JE20080094

**Published:** 2009-07-05

**Authors:** Kaori Ishii, Shigeru Inoue, Yumiko Ohya, Yuko Odagiri, Tomoko Takamiya, Kenichi Suijo, Neville Owen, Teruichi Shimomitsu

**Affiliations:** 1 Tokyo Medical University, Department of Preventive Medicine and Public Health, Tokyo, Japan; 2 Cancer Prevention Research Centre, School of Population Health, The University of Queensland, Brisbane, Australia

**Keywords:** exercise, physical activity, perceived barrier, sociodemographic correlates

## Abstract

**Background:**

The perception of barriers to exercise is an important correlate of exercise participation. However, only a limited number of studies—mostly from Western countries—have attempted to describe the perceptions of barriers to exercise in specific population groups. This study examined the associations between sociodemographic attributes and perceived barriers to exercise in Japanese adults.

**Methods:**

A population-based cross sectional study of 865 participants (age: 20–69 years old, men: 46.5%) was conducted in 4 cities in Japan. Nine sociodemographic attributes (sex, age, location of residence, educational attainment, marital status, employment status, presence of dependents in the household, self-rated health, body mass index), along with exercise frequency and perception of barriers to exercise (discomfort, lack of motivation, lack of time, lack of social support, poor environment) were assessed by self-administered questionnaire.

**Results:**

The most strongly perceived barrier was lack of time. Five of 9 sociodemographic attributes were significantly related to certain types of perceived barriers. Participants who more strongly perceived barriers were younger, more highly educated, more likely to be employed, and had relatively poor self-rated health and a high BMI. The specific types of barriers that were strongly perceived varied with the sociodemographic attributes of the participants.

**Conclusions:**

The results show that the perception of barriers to exercise varies among specific population groups, which indicates the importance of targeting exercise promotion strategies to specific populations.

## INTRODUCTION

Although regular physical activity reduces the risks of morbidity and mortality of diseases such as cardiovascular disease, diabetes, and cancer,^1^ a large proportion of the adult population is not sufficiently physically active to gain these health benefits. In Japan, only 31% of men and 28% of women engage in 30 minutes or more of exercise 2 or more times per week.^2^ A similarly low prevalence of exercisers has been noted in many countries in the world. For example, in the United States less than half the adult population meets the physical activity recommendation to participate in at least 30 minutes of moderately intense physical activity on most days of the week.^3^^,^^4^ Physical activity promotion remains one of the priorities of public health.

*The World Health Organization Guide for Population-based Approaches to Increase Levels of Physical Activity* encourages national action plans, including large-scale interventions to reach the whole population.^5^ This guide also emphasizes that, “Some interventions may be tailored to specific population groups, such as adults, children, older persons, employees, people with disabilities, women, men, cultural groups, and people at risk to develop non-communicable diseases.” To accomplish this, determinants of physical activity among specific population groups must be understood.

Exercise is an important domain of physical activity. Therefore, understanding exercise determinants is a key area of physical activity promotion. Perceptions of barriers to exercise can be important determinants of exercise participation.^6^^–^^8^ Janz et al^9^ indicated that, “Perceived barriers will be strong predictors of behavior change.” According to the health belief model, a person will have a negative attitude toward exercise as a means to promote health when there are more perceived barriers than benefits.^10^ Strategies that consider barriers should be incorporated into interventions to promote exercise.^11^ Thus, the understanding of perceived barriers among specific population groups is important for promoting exercise.

Studies have examined the perception of barriers in convenience samples^12^ and among specific populations, such as students and overweight persons.^8^^,^^13^^–^^16^ However, there have been only a limited number of population-based studies, which were conducted in Europe,^17^ Brazil,^18^ and Australia.^19^ These studies demonstrated that perception of barriers varied according to the sociodemographic attributes of the populations. In addition, the relationships differed by country. Therefore, conducting research in a number of countries should prove useful in better understanding exercise behavior. There are few published studies on sociodemographic variation in perceived barriers to exercise among Japanese.^15^^,^^20^ The present study therefore examined the perception of barriers to exercise in specific population groups among Japanese adults.

## METHODS

### Participants and data collection

In this cross-sectional study, data were collected from February 2007 through January 2008. A total of 4000 residents, aged 20 to 69 years, who lived in 4 Japanese cities (Koganei, Tsukuba, Shizuoka, Kagoshima) were randomly selected from the registry of residential addresses of each city, and stratified by sex (male/female), age (20–29 years, 30–39 years, 40–49 years, 50–59 years, and 60–69 years), and city of residence so that the sample included 2000 subjects of each sex, 800 subjects of each age category, and 1000 subjects from each city. As a result, the addresses of 100 subjects of a specific sex, in a specific age category, and living in a specific city were obtained. Four divergent Japanese cities were chosen in order to account for lifestyle variations. Koganei is a suburban city of Tokyo; Tsukuba is a university town located 50 km northeast of Tokyo; and Shizuoka and Kagoshima are middle-sized cities located in central and west Japan, respectively.

Because of the large number of questions and the use of an accelerometer for other purposes of this project, the survey was divided into 2 parts. Both parts of the survey were conducted by mail. Questionnaires were sent to and collected from participants via post. Participants who agreed to participate in the second survey were sent the materials approximately 7 days after receiving their response to the first survey. Participants were asked to sign the questionnaire before answering. The first survey was a self-administered questionnaire that included questions on sociodemographic status and exercise habits. The second survey consisted of a 7-day accelerometer survey and a second self-administered questionnaire with additional items, which included the scale of perceived barriers to exercise. To obtain a better response rate, participation letters that described the contents of the study were sent to all 4000 subjects 2 weeks before the first survey. During the survey, a call center was set up for subjects who had enquiries regarding the survey. For nonrespondents, requests to join the survey were mailed twice. If the survey was incomplete, we asked the participant to redo the survey. As a result, among the 4000 residents asked to participate, 1508 (37.7%) responded to the first survey; 865 (57.4%) of these 1508 participated in the second survey, which resulted in a final response rate of 21.6%. The response rates for the 4 cities were 20.7% (Tsukuba, 207/1000), 24.8% (Koganei, 248/1000), 22.2% (Shizuoka, 222/1000), and 18.8% (Kagoshima, 188/1000), respectively. In this study, we used data on sociodemographic status and exercise habits from the first survey and data on perceived barriers to exercise from the second survey.

All participants signed an informed consent document before answering the questionnaire. This study received prior approval from the Tokyo Medical University Ethics Committee.

### Measures

#### Perceived barriers to exercise

The Perceived Barriers to Exercise Scale^20^ was the dependent variable. All items of this scale and Cronbach’s alpha coefficients in this study sample are shown in Table 1. The scale consists of 5 subscales: (1) “discomfort,” which comprises 7 items, including “causes sore muscles” and “get hot and sweaty” (Cronbach’s alpha, 0.85), (2) “lack of motivation,” which comprises “too lazy” and “lack of motivation” (0.70), (3) “lack of time,” which comprises 5 items, including “too busy” and “not enough time” (0.85), (4) “lack of social support,” which comprises 4 items, including “family does not encourage” and “friends do not exercise” (0.73), and (5) “poor environment,” which comprises “bad weather” and “lack of facilities” (0.60). Participants provided ratings on a 5-point Likert scale ranging from 1 (strongly disagree) to 5 (strongly agree) to the statement: “When I do not exercise, the important barrier is ...” followed by 20 barrier items. The mean values of the number of selected choices were calculated as scores of factors (range, 1 to 5). A higher score meant a stronger perception of the barrier. The factor structure, reliability, and criterion-related validity of the scale, as compared with the stage of change in exercise behavior, were confirmed in a previous study.^20^

**Table 1.** Scale for perceived barriers to exercise

Factors	Items	Alpha*
Discomfort		0.85
	Causes sore muscles	
	Look silly	
	Too uncoordinated	
	Too boring	
	Get hot and sweaty	
	Too fatigued by exercise	
	Uncomfortable	
Lack of motivation		0.70
	Too lazy	
	Lack of motivation	
Lack of time		0.85
	Too busy	
	Not enough time	
	Too much work to do	
	Interferes with work	
	Too tired	
Lack of social support		0.73
	Family does not encourage	
	Friends do not exercise	
	Interferes with social life	
	No one to exercise with	
Poor environment		0.60
	Bad weather	
	Lack of facilities	

*Cronbach’s coefficient alpha.

#### Sociodemographic attributes and exercise habits

Sex, age, location of residence, educational attainment, employment status, marital status, presence of dependents (living with a child or a person in need of care), self-rated health, body mass index (BMI), and exercise habits were assessed by self-administered questionnaire. In this study, a child was defined as a junior high school student or younger. Self-rated health was measured with a single item that asked participants to rate their health. Participants chose the most suitable answer from a 5-point scale: excellent, very good, good, fair, and poor, for the statement of, “In general, would you say that your health is ...?”. BMI was calculated based on self-reported weight and height. Regular exercise frequency (days/month) in a typical month was queried if the participant engaged in exercise for at least 60 minutes per month.

### Statistical analysis

The Mann–Whitney U test and Kruskal–Wallis test were used to examine differences in perceptions of barriers to exercise by sociodemographic attributes. Responses to sociodemographic attributes and exercise habits were categorized as: sex (men/women), age (20–39 years/40–59 years/60–69 years old), location of residence (Tsukuba/Koganei/Shizuoka/Kagoshima), educational attainment (<13 years/≥13 years), employment status (employed/not employed), marital status (married/not married), presence of dependents (living with a child or a person in need of care/without dependent), self-rated health (good: excellent, very good, or good/fair or poor: fair or poor), BMI (<25.0/≥25.0), and exercise habits (<3 days/week/≥3 days/week). To examine the independent relationships between each sociodemographic variable and perceived barriers to exercise, multiple logistic regression analyses were conducted. For these analyses, all 9 sociodemographic variables were included in the model. Scores for perceived barriers were converted into dichotomous variables at the median. Locations of residences were included in the model as dummy variables. The odds of higher perceived barriers for the 9 sociodemographic attributes (sex, age, location of residence, educational attainment, employment status, marital status, presence of dependents, self-rated health, and BMI) were calculated. A *P* value of less than 0.05 was considered to indicate statistical significance. All statistical analyses were performed with SPSS 12.0J for Windows, SPSS Inc., Chicago, USA.

## RESULTS

### Participant characteristics

Table 2 shows the characteristics of the participants. In the overall sample, 46.5% of the participants were men. The mean age (SD: standard deviation) was 47.9 (13.9) years old. Average BMI was 23.5 (3.0) in men and 21.5 (3.1) in women. The percentage of regular exercisers (≥3 days/week) was 19.4% in men and 22.3% in women. The characteristics of participants living in each city are also shown in Table 2. The prevalence of exercisers and 3 sociodemographic variables—educational attainment, employment status, and living status—significantly differed by city.

**Table 2.** Descriptive characteristics (numbers and percentages) of subjects and subsamples

	Overall	Men	Women	*P* value*	Tsukuba	Koganei	Shizuoka	Kagoshima	*P* value^†^
	*n* = 865	*n* = 403	*n* = 462		*n* = 207	*n* = 248	*n* = 222	*n* = 188	
							
	*n* (%)	*n* (%)	*n* (%)		*n* (%)	*n* (%)	*n* (%)	*n* (%)	
Age, years									
​ 20–29	135 (15.6)	52 (12.9)	83 (18.0)		37 (17.9)	36 (14.5)	31 (14.0)	31 (16.5)	
​ 30–39	140 (16.2)	53 (13.2)	87 (18.8)		36 (17.4)	40 (16.1)	33 (14.9)	31 (16.5)	
​ 40–49	185 (21.4)	85 (21.1)	100 (21.6)		48 (23.2)	50 (20.2)	53 (23.9)	34 (18.1)	
​ 50–59	188 (21.7)	104 (25.8)	84 (18.2)		43 (20.8)	51 (20.6)	51 (23.0)	43 (22.9)	
​ 60–69	217 (25.1)	109 (27.0)	108 (23.4)		43 (20.8)	71 (28.6)	54 (24.3)	49 (26.1)	
​ Mean ± SD	47.9 ± 13.9	49.3 ± 13.5	46.6 ± 14.3	0.001	45.5 ± 13.7	48.7 ± 14.1	48.3 ± 13.9	47.3 ± 14.3	0.090

Education, years									
​ <13	332 (38.6)	148 (37.1)	184 (39.8)	0.440	74 (35.9)	62 (25.2)	101 (45.5)	95 (50.8)	<0.001
​ ≥13	529 (61.4)	251 (62.9)	278 (60.2)		132 (64.1)	184 (74.8)	121 (54.5)	92 (49.2)	

Marital status									
​ Married	650 (75.5)	321 (79.9)	329 (71.7)	0.005	153 (74.6)	183 (74.1)	179 (81.0)	135 (71.8)	0.148
​ Not married	211 (24.5)	81 (20.1)	130 (28.3)		52 (25.4)	64 (25.9)	42 (19.0)	53 (28.2)	

Employment status									
​ Employed	644 (74.5)	345 (85.6)	299 (64.9)	<0.001	169 (81.6)	179 (72.5)	172 (77.5)	124 (66.0)	0.002
​ Not employed	220 (25.5)	58 (14.4)	162 (35.1)		38 (18.4)	68 (27.5)	50 (22.5)	64 (34.0)	

Living with a child or person in need of care									
​ Yes	313 (36.2)	136 (33.8)	177 (38.3)	0.178	87 (42.0)	75 (30.4)	94 (42.3)	57 (30.3)	0.004
​ No	551 (63.8)	266 (66.2)	285 (61.7)		120 (58.0)	172 (69.6)	128 (57.7)	131 (69.7)	

Self-rated health									
​ Fair or poor	407 (47.2)	205 (50.9)	202 (43.9)	0.047	98 (47.8)	117 (47.2)	111 (50.0)	81 (43.1)	0.571
​ Good	456 (52.8)	198 (49.1)	258 (56.1)		107 (52.2)	131 (52.8)	111 (50.0)	107 (56.9)	

BMI, kg/m^2^									
​ <25	689 (79.9)	288 (71.6)	401 (87.2)		161 (77.8)	204 (82.6)	181 (81.5)	143 (76.9)	
​ ≥25	173 (20.1)	114 (28.4)	59 (12.8)		46 (22.2)	43 (17.4)	41 (18.5)	43 (23.1)	
​ Mean ± SD	22.4 ± 3.2	23.5 ± 3.0	21.5 ± 3.1	<0.001	22.5 ± 3.3	22.3 ± 3.2	22.4 ± 3.2	22.4 ± 3.2	0.926

Exercise, days/week									
​ <3	684 (79.1)	325 (80.6)	359 (77.7)	0.315	156 (75.4)	202 (81.5)	163 (73.4)	163 (86.7)	0.004
​ ≥3	181 (20.9)	78 (19.4)	103 (22.3)		51 (24.6)	46 (18.5)	59 (26.6)	25 (13.3)	

Abbreviations: BMI, body mass index; SD, standard deviation.

*Comparisons between men and women, using the chi-square test or *t*-test.

^†^Comparisons between locations of residence, using the chi-square test or ANOVA.

The total numbers of respondents are not always equal, due to missing data.

### Associations between perceived barriers and sociodemographic attributes

Among the overall sample, the medians (25th percentile to 75th percentile) of barrier scores were lack of time 3.0 (2.3–3.8), lack of motivation 2.9 (2.1–3.7), poor environment 2.2 (1.4–3.0), discomfort 1.8 (1.3–2.4), and lack of social support 1.6 (1.1–2.3) (Table 3). Perceptions of barriers to exercise differed significantly for 8 of 9 sociodemographic attributes and by exercise habit. Only location of residence was not related to perceived barriers. Men perceived significantly stronger barriers to exercise, as did participants who were younger, more highly educated, not married, employed, living with a child or person in need of care, overweight, nonexercisers, and had poorer self-rated health. Subscales of perceived barriers related to these variables differed by sociodemographic attributes. For example, subscales related to age were lack of motivation and lack of time, while those related to BMI were discomfort, lack of social support, and poor environment.

**Table 3.** Comparison of scores for perceived barriers to exercise, by sociodemographic variables and exercise habits

	n*	Discomfort	Lack of motivation	Lack of time	Lack of social support	Poor environment
					
		Median (25%–75%)^†^	Median (25%–75%)^†^	Median (25%–75%)^†^	Median (25%–75%)^†^	Median (25%–75%)^†^
Overall		1.8 (1.3–2.4)	2.9 (2.1–3.7)	3.0 (2.3–3.8)	1.6 (1.1–2.3)	2.2 (1.4–3.0)

Sex						
​ Male	403	2.0 (1.4–2.5)	2.9 (2.1–3.7)	3.0 (2.3–3.8)	1.8 (1.1–2.4)	2.3 (1.5–3.0)
​ Female	462	1.7 (1.3–2.4)	3.0 (2.2–3.6)	3.1 (2.3–3.8)	1.6 (1.1–2.2)	2.2 (1.4–3.0)
​ ​ *P* value^‡^		0.028	0.944	0.952	0.102	0.282

Age, years						
​ 20–39	275	1.8 (1.3–2.5)	3.1 (2.3–3.9)	3.3 (2.6–3.9)	1.6 (1.1–2.3)	2.3 (1.4–3.1)
​ 40–59	373	1.9 (1.4–2.5)	3.0 (2.2–3.7)	3.2 (2.5–3.9)	1.6 (1.1–2.3)	2.1 (1.4–2.9)
​ 60–69	217	1.8 (1.3–2.4)	2.6 (1.8–3.3)	2.4 (1.7–3.2)	1.6 (1.1–2.3)	2.4 (1.5–3.0)
​ ​ *P* value^§^		0.320	<0.001	<0.001	0.706	0.155

Location of residence						
​ Tsukuba	207	1.8 (1.3–2.5)	2.9 (2.1–3.7)	3.0 (2.3–3.9)	1.6 (1.1–2.3)	2.2 (1.4–3.0)
​ Koganei	248	1.8 (1.4–2.4)	3.0 (2.3–3.8)	3.1 (2.5–3.7)	1.6 (1.1–2.2)	2.3 (1.6–3.0)
​ Shizuoka	222	1.9 (1.3–2.5)	3.0 (2.2–3.6)	3.0 (2.2–3.9)	1.7 (1.1–2.4)	2.3 (1.5–3.0)
​ Kagoshima	188	1.8 (1.3–2.3)	2.8 (2.0–3.5)	2.9 (2.1–3.6)	1.7 (1.1–2.3)	2.1 (1.3–2.9)
​ ​ *P* value^§^		0.501	0.203	0.149	0.603	0.215

Education, years						
​ <13	332	1.8 (1.4–2.5)	2.7 (1.9–3.4)	2.8 (2.1–3.6)	1.7 (1.1–2.3)	2.2 (1.4–3.0)
​ ≥13	529	1.8 (1.3–2.4)	3.1 (2.3–3.8)	3.2 (2.4–3.8)	1.6 (1.1–2.3)	2.2 (1.5–3.0)
​ ​ *P* value^‡^		0.751	<0.001	0.001	0.193	0.979

Marital status						
​ Married	650	1.9 (1.3–2.4)	2.9 (2.1–3.6)	3.0 (2.3–3.7)	1.6 (1.1–2.2)	2.2 (1.4–3.0)
​ Not married	211	1.8 (1.3–2.6)	3.0 (2.2–3.8)	3.2 (2.4–4.0)	1.7 (1.1–2.5)	2.3 (1.4–3.1)
​ ​ *P* value^‡^		0.515	0.491	0.020	0.344	0.594

Employment status						
​ Employed	644	1.8 (1.3–2.5)	3.0 (2.2–3.7)	3.2 (2.5–3.9)	1.6 (1.1–2.3)	2.2 (1.5–3.0)
​ Not employed	220	1.8 (1.3–2.4)	2.9 (2.0–3.5)	2.5 (1.7–3.2)	1.6 (1.1–2.3)	2.2 (1.4–3.0)
​ ​ *P* value^‡^		0.600	0.317	<0.001	0.832	0.740

Living with a child or person in need of care						
​ Yes	313	1.7 (1.3–2.5)	2.9 (2.0–3.7)	3.2 (2.4–3.9)	1.6 (1.1–2.3)	2.2 (1.4–3.0)
​ No	551	1.9 (1.4–2.4)	3.0 (2.2–3.7)	3.0 (2.2–3.6)	1.6 (1.1–2.3)	2.2 (1.5–3.0)
​ ​ *P* value^‡^		0.301	0.473	0.010	0.941	0.944

Self-rated health						
​ Fair or poor	407	2.1 (1.5–2.6)	3.1 (2.3–3.8)	3.0 (2.3–3.8)	1.8 (1.1–2.4)	2.3 (1.6–3.0)
​ Good	456	1.7 (1.2–2.3)	2.8 (2.0–3.5)	3.1 (2.3–3.8)	1.5 (1.1–2.2)	2.1 (1.3–3.0)
​ ​ *P* value^‡^		<0.001	<0.001	0.849	0.008	0.037

BMI, kg/m^2^						
​ <25	689	1.8 (1.3–2.4)	2.9 (2.1–3.7)	3.0 (2.3–3.7)	1.5 (1.1–2.3)	2.2 (1.4–2.9)
​ ≥25	173	2.1 (1.4–2.6)	3.1 (2.4–3.7)	3.1 (2.2–3.9)	1.9 (1.2–2.4)	2.5 (1.7–3.3)
​ ​ *P* value^‡^		0.010	0.169	0.732	0.028	0.001

Exercise, days/week						
​ <3	684	1.9 (1.4–2.5)	3.0 (2.2–3.7)	3.1 (2.4–3.9)	1.7 (1.1–2.3)	2.3 (1.5–3.0)
​ ≥3	181	1.5 (1.1–2.3)	2.7 (1.7–3.4)	2.8 (1.9–3.5)	1.3 (1.0–2.1)	2.2 (1.3–3.0)
​ ​ *P* value^‡^		<0.001	<0.001	0.001	0.001	0.519

Abbreviation: BMI, body mass index.

Higher score means higher perception of a barrier to exercise.

*Total numbers of respondents are not equal, due to missing data.

^†^Twenty-fifth and 75th percentiles.

^‡^The Mann–Whitney U test was used to compare the scores of barrier perception between groups.

^§^The Kruskal–Wallis test was used to compare the scores of barrier perception between groups.

Table 4 shows the odds ratios of participants who perceived higher barriers. According to the results, 5 of 9 variables—age, education, employment status, self-rated health, and BMI—were independently related to the perception of barriers. Sex, location of residence, marital status, and presence of dependents were not associated with barrier perception. Younger participants perceived lack of motivation and lack of time more strongly than did older participants. Middle-aged participants perceived poor environment as a less of a barrier than did those who were older (60–69 years old). As for employment status, employed participants strongly perceived lack of time. Poorer self-rated health was significantly related to strong perceptions of discomfort, lack of motivation, and lack of social support. Regarding BMI, overweight participants (≥25.0) perceived stronger barriers of discomfort, lack of social support, and poor environment.

**Table 4.** Odds ratios and 95% confidence intervals for participants who perceived higher barriers to exercise, after evaluation by multiple logistic regression analyses

	Discomfort			Lack of motivation			Lack of time			Lack of social support			Poor environment		
					
	OR	95% CI	*P* value	OR	95% CI	*P* value	OR	95% CI	*P* value	OR	95% CI	*P* value	OR	95% CI	*P* value
Sex															
​ Male	1.32	0.95–1.84	0.096	1.06	0.75–1.49	0.749	0.78	0.55–1.09	0.148	1.22	0.88–1.69	0.241	1.08	0.78–1.50	0.649
​ Female	1.00			1.00			1.00			1.00			1.00		

Age, years															
​ 20–39	1.13	0.68–1.86	0.640	2.31	1.34–3.99	0.003	2.52	1.47–4.32	0.001	1.11	0.67–1.82	0.693	1.01	0.61–1.67	0.955
​ 40–59	1.22	0.79–1.89	0.375	1.74	1.08–2.82	0.023	2.22	1.39–3.56	0.001	1.00	0.64–1.54	0.983	0.56	0.36–0.87	0.010
​ 60–	1.00			1.00			1.00			1.00			1.00		

Location of residence															
​ Tsukuba	0.92	0.57–1.47	0.712	1.05	0.64–1.74	0.842	0.97	0.59–1.58	0.897	1.13	0.70–1.80	0.620	0.92	0.57–1.48	0.735
​ Koganei	0.79	0.50–1.24	0.302	0.81	0.50–1.31	0.393	0.78	0.49–1.26	0.312	1.03	0.65–1.62	0.907	0.66	0.41–1.04	0.072
​ Shizuoka	0.72	0.45–1.15	0.173	0.90	0.55–1.48	0.676	0.88	0.54–1.43	0.609	0.85	0.54–1.35	0.495	0.81	0.51–1.28	0.361
​ Kagoshima	1.00			1.00			1.00			1.00			1.00		

Education, years															
​ ≥13	0.99	0.70–1.40	0.959	1.63	1.13–2.34	0.009	1.29	0.91–1.84	0.158	0.84	0.59–1.18	0.304	0.95	0.67–1.33	0.747
​ <13	1.00			1.00			1.00			1.00			1.00		

Marital status															
​ Not married	0.93	0.61–1.41	0.724	0.85	0.55–1.33	0.487	1.21	0.78–1.88	0.386	1.03	0.67–1.56	0.908	0.93	0.61–1.41	0.728
​ Married	1.00			1.00			1.00			1.00			1.00		

Employment status															
​ Employed	0.87	0.58–1.29	0.478	0.96	0.63–1.47	0.866	2.77	1.81–4.25	<0.001	0.97	0.65–1.43	0.862	1.07	0.72–1.59	0.752
​ Not employed	1.00			1.00			1.00			1.00			1.00		

Living with a child or person in need of care															
​ Yes	0.73	0.51–1.03	0.072	0.86	0.60–1.24	0.425	1.20	0.84–1.72	0.307	0.98	0.69–1.38	0.889	1.09	0.77–1.54	0.634
​ No	1.00			1.00			1.00			1.00			1.00		

Self-rated health															
​ Fair or poor	1.64	1.20–2.25	0.002	1.88	1.35–2.61	<0.001	1.03	0.74–1.42	0.881	1.42	1.04–1.93	0.029	1.19	0.87–1.63	0.270
​ Good	1.00			1.00			1.00			1.00			1.00		

BMI, kg/m^2^															
​ ≥25	1.53	1.03–2.27	0.037	1.08	0.72–1.62	0.712	1.04	0.69–1.56	0.865	1.90	1.28–2.82	0.001	1.79	1.21–2.65	0.004
​ <25	1.00			1.00			1.00			1.00			1.00		

Abbreviations: BMI, Body mass index; CI, confidence interval; OR, odds ratio.

Odds ratios were calculated after adjustment for all variables listed in the table.

## DISCUSSION

This study examined specific barriers to exercise perceived by populations of Japanese adults characterized by 9 sociodemographic attributes. The results indicated that 5 of 9 sociodemographic attributes were independently related to perception of barriers. In general, those who perceived higher barriers were younger, more highly educated, employed, had fair or poor self-rated health, and a high BMI. The specific types of perceived barriers varied by sociodemographic characteristics. For example, age was related to lack of motivation, lack of time, and poor environment, but not to other barriers. As for BMI, discomfort, lack of social support, and poor environment were more strongly perceived among overweight participants. Additional associations between population characteristics and specific types of barriers were also revealed in this study. These findings are important to better understand the correlates of exercise habits among specific population groups, and have implications for the development of exercise promotion strategies that are adjusted to the needs of target populations.

Among all participants, lack of time was the barrier for which the median was highest. As compared to 3 studies from other countries using population-based samples, our results are similar to those of the European^17^ and Australian^19^ studies, but not to those of the Brazilian study.^18^ Because these studies used different scales to measure barriers, comparison and interpretation of the results must be undertaken carefully. Depending on the wording of items in each study, work/study, no time, and lack of time were the strongest barriers, respectively, in the European, Australian, and the present study, while in the Brazilian study, lack of money was reported as the strongest barrier. Lack of time was fourth-ranked among the 8 barriers in the Brazilian study. Regarding the relationship between sociodemographic characteristics and this barrier, all studies reported that lack of time was perceived more strongly among younger, as compared to older, age groups. However, the relationship between sex and the lack of time barrier is more complicated. In the European study, men strongly perceived this barrier; however, it was perceived more strongly among women in Brazil. By contrast, there were no sex differences in the perception of time constraints in either the present study or the Australian study. This suggests that the associations between specific types of barriers and population characteristics vary according to cultural background. Employment status was also related to lack of time in the present study. For those who have little discretionary time for exercise, time-saving interventions such as lifestyle intervention rather than structured exercise programs, internet programs rather than face-to-face counseling, and individual counseling rather than group programs may be more effective.

Discomfort was significantly associated with fair or poor self-rated health and overweight, which suggests that exercise programs of proper intensity that are adjusted to a participant’s fitness level and do not induce discomfort such as muscle soreness may be effective among individuals who are overweight or in poor health. Other relationships between sociodemographic variables and barriers indicated that certain population groups have their own profile of barrier perception.

This study was conducted using a community-based random sample from residents living in 4 cities in Japan. Participants were randomly selected from the registry of residential addresses, from a list encompassing the entire population of each city. The response rate was 21.6%. One reason for this low rate was that we included a 7-day accelerometer survey for other purposes of this project. To estimate the representativeness of this sample, we compared the age-adjusted prevalence of overweight individuals, exercisers, and employees in our sample with those in the national survey. The prevalence of overweight participants (BMI ≥25.0) was 28.4% in men and 12.8% in women in this study, while the age-adjusted prevalence in the sample of the Japanese National Health and Nutrition Survey 2005^2^ was 29.6% in men and 19.0% in women. The prevalence of overweight persons in this study was similar in men and 6.2% lower in women. There are 2 possible reasons for this lower prevalence of overweight among women. One is that this sample of women was relatively healthier than the general population of Japanese. Another is that the assessment of BMI in this study was based on self-reports and women tend to report a lower weight than their actual body weight.^21^^,^^22^ As for exercise habits (≥3days/week), 19.4% of men and 22.3% of women in this study were exercisers, while 20.4% of men and 18.2% of women were reported to be exercisers (≥3days/week) in the national survey.^2^ Although the survey methods were different, the sample of this study seems to include a slightly higher percentage of women exercisers. Regarding employment status, 85.6% of men and 64.9% of women in the present study had gainful employment, while 76.3% of men and 55.2% of women worked full-time or part-time in the national survey.^23^ The participants of this study may have included more employed persons, which indicates that our sample was slightly different from the general population. Therefore, we cannot exclude the possibility of selection bias. This study sample may have been slightly healthier and higher in socioeconomic status. If we assume that this bias in our population results in behavioral skills that are more likely to overcome the actual barriers caused by sociodemographic status, as compared to an unhealthier population with lower socioeconomic status, then this study would underestimate the influence of sociodemographic status on barrier perceptions.

This study possesses several strengths. First, we used a sample from the general population, whereas most previous studies of perceived barriers were conducted using certain populations such as students, employees, and research volunteers. Therefore, this study contributes to the understanding of the perception of barriers in the general population, and the difference in barriers among specific population groups. Second, most studies of exercise barriers were conducted in Western countries. There have been few reports from Japan, which has important cultural differences from Western countries. Our results have implications for exercise promotion strategies developed specifically for Japanese. Third, our analysis integrated a large variety of sociodemographic attributes. Most studies examined barriers by sex, age, and a few other attributes such as BMI and employment status, but the present study investigated a larger number of attributes. Finally, the reliability and validity of the barrier scale were comprehensively examined and confirmed in a previous study.^20^

However, some limitations of the present study should be acknowledged. First, the response rate was relatively low. As discussed above, this low rate could result in selection bias. However, there have been few previous studies that have addressed perceived barriers to exercise, with respect to a variety of sociodemographic variables, among the general population. Thus, we believe that our results are useful for understanding the psychological aspects of exercise behavior. Second, we examined barriers to exercise, but did not investigate other domains of physical activity such as work activity, commuting, and household work, which are also beneficial to health. The perception of barriers to exercise and to other domains of physical activity may be different. In the future, research on barriers to specific domains of physical activity would be useful in understanding determinants of physical activity.

In spite of these limitations, the results of this study helped to identify subgroups that perceive specific barriers to exercise among Japanese, and to gain a better understanding of the psychological aspects of exercise behavior. Characteristics of specific population groups should be considered in the development of exercise promotion strategies.

## CONCLUSION

Perception of barriers to exercise varied in a Japanese population characterized by age, educational attainment, employment status, self-rated health, and BMI. These results should prove helpful in developing population-specific interventions, such as time-saving interventions for younger and employed populations and, for groups with poorer health, exercise programs that are adjusted to the participants’ fitness level. The results of this study highlight the importance of adjusting exercise promotion strategies to match the characteristics of the target population.
